# Incidence, associated factors, and outcomes of delirium in critically ill children in china: a prospective cohort study

**DOI:** 10.1186/s12888-023-05406-3

**Published:** 2023-12-11

**Authors:** Lei Lei, Yi Li, Huilin Xu, Qin Zhang, Jiacai Wu, Shoujv Zhao, Xiaochao Zhang, Min Xu, Shuai Zhang

**Affiliations:** 1 Department of Pediatric Intensive Care Unit Nursing, West China Second University Hospital, Sichuan University, West China School of Nursing, Sichuan University, No. 20, Section 3, South Renmin Road, Chengdu, Sichuan China; 2 Key Laboratory of Birth Defects and Related Diseases of Women and Children (Sichuan University), Ministry of Education, Chengdu, Sichuan China

**Keywords:** Pediatrics, Critical care, Delirium, Southwest China

## Abstract

**Background:**

Delirium occurs frequently in critically ill children and has been reported in many countries, but delirium is not well-characterized in China. The aim of this study was to represent the incidence of delirium in critically ill children in China, its associated factors, and the influence of delirium on in-hospital outcomes.

**Methods:**

This observational prospective cohort study was set up in a large academic medical center with a 57-bed PICU in southwestern China. Critically ill children who required PICU stays over 24 h and were admitted between November 2019 and February 2022 were included in this study. The Cornell Assessment of Pediatric Delirium was used twice daily for delirium evaluation by bedside nurses, and twenty-four clinical features were collected from medical and nursing records during hospitalization.

**Results:**

The incidence of delirium was 26.0% (*n* = 410/1576). Multivariate analysis revealed that seven independent risk factors including days of mechanical ventilation and physical restraints, admission diagnosis (neurologic disorder), sleep deprivation, use of benzodiazepines and dexmedetomidine, liver failure/liver dysfunction associated with delirium in critically ill children. One potentially protective factor was the watching television /listening to music/playing with toys. Children with delirium had longer lengths of stay in the PICU (median 11 vs. 10 days, *p* < 0.001) and hospital (median 18 vs. 15 days, *p* < 0.001) compared to those without delirium. Additionally, the in-hospital mortality rates were 4.63% and 0.77% in patients with and without delirium (*p* < 0.05).

**Conclusions:**

Delirium is common in critically ill children in China and related to poor outcomes. Interventional studies are warranted to determine the best practices to reduce delirium exposure in at-risk children.

## Introduction

Delirium is defined as an altered mental state characterized by an acute-onset, fluctuating course of disturbed awareness and cognition [1]. It is a neuropsychiatric condition secondary to a general medical condition and/or its treatments [2]. In critically ill children, delirium occurs with reported rates ranging from 12 to 65% [3], and from 22.3% to 54.7% in China specifically [4–7]. However, due to the lack of routine screening, there is no large sample size report that describes the incidence of delirium in critically ill children in China. In fact, 80.4% of healthcare professionals in China do not perform daily screening for delirium in pediatric intensive care units (PICUs) [8]. Delirium has been extensively studied and reported to be associated with adverse outcomes, including increased mortality rates, longer hospital stays, prolonged duration of invasive mechanical ventilation, higher hospitalization costs, and an even greater risk of decreased quality of life after discharge from the PICU [9–12]. Despite this, there is still a lack of data regarding basic outcomes in critically ill children who develop delirium in China.

The exact pathophysiological cause of delirium is currently unclear, so exploration of the related influencing factors remains the basis for current delirium intervention, especially the exploration of protective factors. The most recent meta-analysis showed seven frequent risk factors related to delirium in critically ill children, including developmental delay, mechanical ventilation, physical restraints, and receiving either benzodiazepines, opiates, steroids, or vasoactive medication [13]. However, little is known about the accuracy of the protective factors of delirium, and most of the studies on the prevention of delirium focus on a nonpharmacological delirium bundle of interventions to manage delirium in PICU patients. The key concepts for those interventions are a regular assessment throughout the PICU stay and the reduction of modifiable risk factors that include restraints, immobility, and benzodiazepines use, as well as creating the conditions for nocturnal sleep and healthy circadian rhythms and encouraging family participation [14–17].

Given the lack of specific data on delirium in critically ill Chinese children, developing localized delirium intervention strategies is difficult. Therefore, our aims with this study were to represent the incidence of delirium in critically ill children in China, and determine the medical and nursing-related risk factors for its development. Additionally, we aimed to compare in-hospital mortality rates and lengths of stay in both the PICU and hospital between children with and without delirium.

## Methods

### Study setting and participants

An observational prospective study was conducted in a large tertiary academic hospital with a 57-bed mixed PICU in southwestern China between November, 2019 and February, 2022. Ethics approval was obtained from the Medical Ethics Committee of West China Second Hospital of Sichuan University (No. 2020084), and written informed consent was obtained from all participants’ legal surrogates or parents in this trial. Children were excluded if they: had a PICU stay of less than 24 h, were delirious upon admission to the PICU(the score of the Cornell Assessment of Pediatric Delirium was 10 or higher at admission assessment), left treatment at the discretion of their guardian, could not be reliably assessed for delirium (e.g., sustained a coma throughout their entire PICU stay, or had clearly diagnosed mental illness, auditory or visual disorders, or myasthenia), or if the completion rate for delirium screening was less than 90% during an individual child’s PICU stay for any reason.

### Delirium assessment

All children in the study were assessed for delirium twice a day (at 9:00am and 5:00 pm during their stay in the PICU) by bedside nurses using the Cornell Assessment of Pediatric Delirium (CAPD). If a patient was receiving sedation, the CAPD was administered as soon as possible following sedation interruption. The CAPD is a valid and reliable monitoring tool for nurses to screen critically ill children of all ages for delirium and takes less than two minutes per patient. It is highly recommended by many guidelines and has been applied worldwide [18–22]. The assessment consists of eight questions, each with a scale of 0 to 4 points. The CAPD score can range from 0 to 32 points, with a cutoff score of ≥ 10 indicating the presence of delirium, and higher scores reflecting more severe symptoms. The translated Chinese version of the CAPD has demonstrated acceptable reliability, and sustainability, with excellent ongoing compliance [23].

All participating nurses received standardized information and guidance on the accurate application of the CAPD method to evaluate the development of delirium. To reduce bias in delirium assessment, quality control measures were implemented. For each study day, the quality control nurse randomly selected half of the patients who had already been assessed by the bedside nurse for reassessment. If there was a significant difference between the two assessments, a psychiatrist was consulted for a third assessment. To reduce potential source of bias, bedside nurses were not informed that the data was being collected for our study.

### Demographic data and clinical records

Twenty-four total possible influencing factors associated with delirium from medical and nursing records during hospitalization were recorded. Thirteen clinical variables included: sex, age (month age), admission diagnosis category (respiratory insufficiency/failure, infectious/inflammatory, neurological disorder, hematologic/oncologic disorder, renal/metabolic disorder, cardiac disease, surgery, others), developmental delay, cognitive or motor dysfunction, urgent admission, metabolic acidosis, liver failure/liver dysfunction, hyperbilirubinemia, days of mechanical ventilation, blood transfusion, days of physical restraints, and sleep deprivation. Eight pharmacological variables were included: treatment with corticosteroids, benzodiazepines, dexmedetomidine, barbiturates, propofol, anticholinergic drugs, opioids, or vasoactive drugs. Finally, three nonpharmacological variables were also included: familiar items, watching television/listening to music/playing with toys (WT/LM/PT), and keeping a diary/drawing a picture (KD/DP). These data were collected by bedside nurses who were blinded to the purpose of the collection.

### Statistical analysis

Statistical analysis was conducted using the statistical software package SPSS V.26.0 for Windows. The data in this study did not conform to normal distributions. Prior to the analysis continuous data were expressed as median (interquartile range, IQR), and categorical variables were expressed as numbers (percentages). In univariate analysis, we compared categorical variables between the delirium cohort and the non-delirium cohort using Pearson's chi-squared test for tables larger than 2 by 2 and compared continuous variables between the two groups using the Mann–Whitney U test. All tests used a two-sided alternative, and *p*-values < 0.05 were considered to be statistically significant. Multivariate logistic regression was then applied to evaluate multivariate associations with delirium. Variables included in the final multivariate model are displayed with odds ratios (OR) and their associated 95% confidence intervals.

## Results

### Sample characteristics

**Fig. 1:**
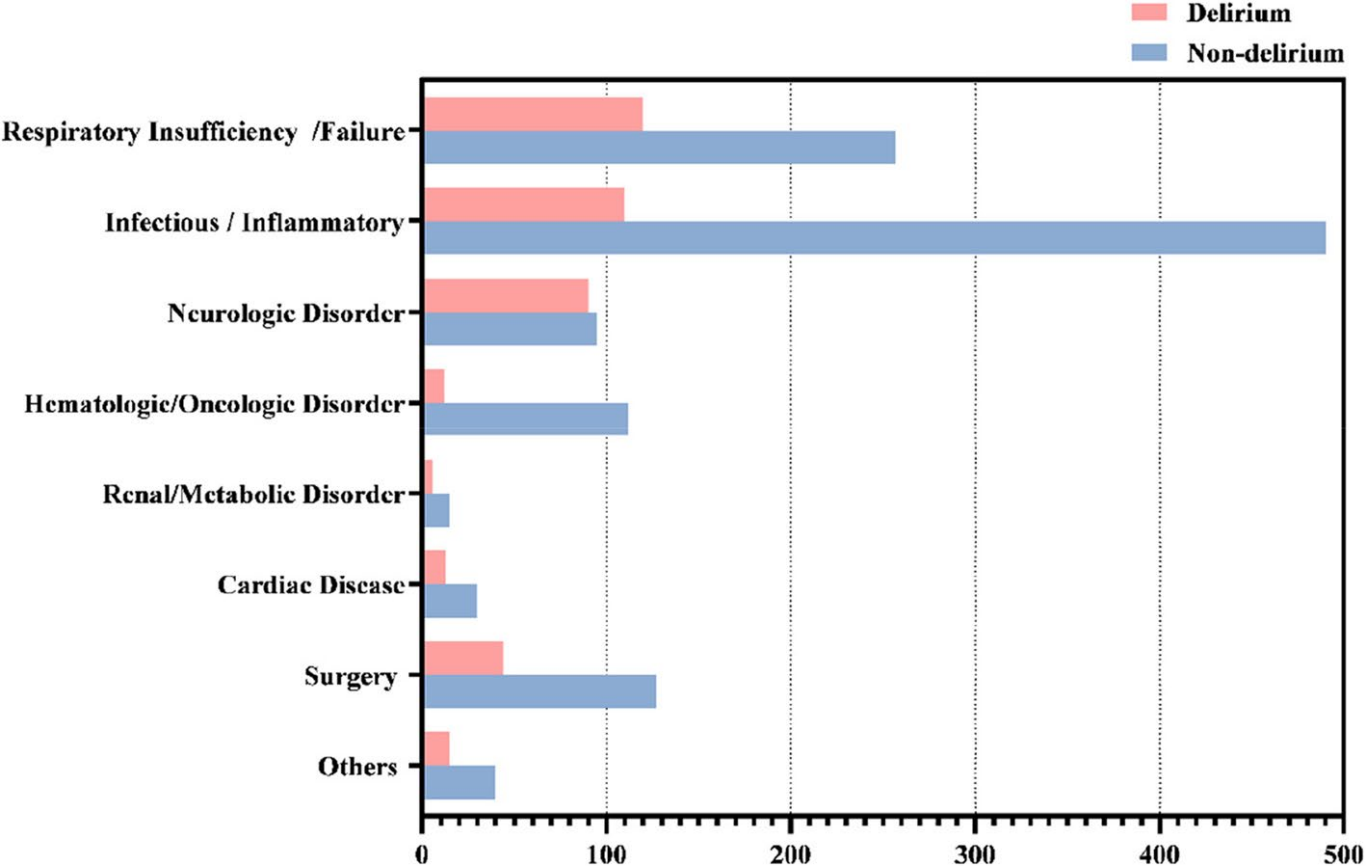
The incidence of delirium varied for different admission diagnoses

A total of 1576 critically ill children were included in this study. Study patients comprised 929(58.9%) males, and the ages of all patients ranged from 28 days to 15 years with a median age of 12 months (IQR 3 to 60 months). Delirium was found in 410(26.0%) of the 1,576 patients. The incidence of delirium varied for different admission diagnoses: respiratory insufficiency/failure (120/377) was 31.8%, infectious/inflammatory (110/600) was 18.3%, neurological disorder (90/185) was 48.6%, hematological/oncological disorder (12/124) was 9.7%, renal/metabolic disorder (6/21) was 28.6%, cardiac disease (13/43) was 30.2%, surgery (44/171) was 25.7%, and all others (15/55) were 27.3% (Fig. 1). In addition, 18.1% of patients were mechanically ventilated, 47.0% of patients experienced at least once physical restraint, and a total 1,853 and 5,002 days of mechanical ventilation and physical restraints were recorded for each of these respectively. The details can be found the Table 1, Fig. 1 and Table 2. In Fig. 1, the X-axis represents the number of patients, and Y-axis represents the classification of admission diagnoses.

**Table 1:** Results of univariate analysis of categorical data

**Characteristic**	**All admissions** (*n* = 1576)	**Delirium**		***P* Value**
		**No**(*n* = 1166)	**Yes**(*n* = 410)	
Gender(n,%)				
Male	929 (58.9)	706 (76.0)	223 (24.0)	0.029
Female	647(41.1)	460(71.1)	187(28.9)	
Cognitive or motor dysfunction(n,%)				
Yes	85 (5.4)	43(50.6)	42 (49.4)	< 0.001
No	1,491 (94.6)	1123 (75.3)	368 (24.7)	
Developmental delay(n,%)				
Yes	102 (6.5)	61(59.8)	41 (40.2)	< 0.001
No	1,474 (93.5)	1105 (75.0)	369 (25.0)	
Urgent admission(n,%)				
Yes	1,319 (83.7)	993 (75.3)	326 (24.7)	< 0.001
No	257 (16.3)	173 (67.3)	84 (32.7)	
Metabolic acidosis(n,%)				
Yes	163 (10.3)	103 (63.2)	60 (36.8)	< 0.001
No	1,413 (89.7)	1063 (75.2)	350 (24.8)	
Liver failure/dysfunction(n,%)				
Yes	345 (21.9)	237 (68.7)	108 (31.3)	0.011
No	1,231 (78.1)	929 (75.5)	302 (24.5)	
Hyperbilirubinemia(n,%)				
Yes	59 (3.7)	44 (74.6)	15 (25.4)	0.916
No	1,517 (96.3)	1,122 (74.0)	395 (26.0)	
Blood transfusion(n,%)				
Yes	435 (27.6)	304 (69.9)	131 (30.1)	0.022
No	1,141 (72.4)	862 (75.5)	279 (24.5)	
Sleep deprivation(n,%)				
Yes	136 (8.6)	74 (54.4)	62 (45.6)	< 0.001
No	1,440 (91.4)	1092 (75.8)	348 (24.2)	
Corticosteroids(n,%)^a^				
Yes	515 (32.7)	332 (64.5)	183 (35.5)	< 0.001
No	1,061 (67.3)	834 (78.6)	227 (21.4)	
Benzodiazepines (n,%)^a^				
Yes	677 (43.0)	375 (55.4)	302 (44.6)	< 0.001
No	899 (57.0)	791 (88.0)	108(12.0)	
Dexmedetomidine (n,%)^a^				
Yes	249 (15.8)	115 (46.2)	134 (53.8)	< 0.001
No	1,327 (84.2)	1051 (79.2)	276 (20.8)	
Barbiturates (n,%)^a^				
Yes	183 (11.6)	96 (52.5)	87 (47.5)	< 0.001
No	1,393 (88.4)	1070 (76.8)	323 (23.2)	
Propofol (n,%)^a^				
Yes	166 (10.5)	63 (38.0)	103 (62.0)	< 0.001
No	1,410 (89.5)	1103 (78.2)	307 (21.8)	
Anticholinergic drugs (n,%)^a^				
Yes	35 (2.2)	18(51.4)	17 (48.6)	< 0.001
No	1,541 (97.8)	1148 (74.5)	393 (25.5)	
Opioids (n,%)^a^				
Yes	311 (19.7)	136 (43.7)	175 (56.3)	< 0.001
No	1,265 (80.3)	1030 (81.4)	235 (18.6)	
Vasoactive drugs(n,%)^a^				
Yes	211 (13.4)	126 (59.7)	85 (40.3)	< 0.001
No	1,365 (86.6)	1040 (76.2)	325 (23.8)	
Familiar items(n,%)				
Yes	105 (6.7)	80 (76.2)	25 (23.8)	0.594
No	1,471 (93.3)	1,086 (73.8)	385 (26.2)	
Watching television /listening to music/playing with toys (WT/LM/PT)(n,%)				
Yes	431 (27.3)	346 (80.3)	85(19.7)	< 0.001
No	1,145 (72.7)	820 (71.6)	325 (28.4)	
Keeping a diary/drawing pictures (KD/DP) (n,%)				
Yes	92 (5.8)	83 (90.2)	9 (9.8)	< 0.001
No	1,484 (94.2)	1,083 (73.0)	401 (27.0)	

^a^For medication categories, “No” and “Yes” indicate whether the patient ever received this class of drug during hospitalization

**Table 2:** Results of univariate analysis of continuous data

**Characteristic**	**All admissions** (*n* = 1576)	**Delirium**		***P*** **Value**
		**No**(*n *= 1166)	**Yes**(*n* = 410)	
Age [months, medium (IQR)]				
	12(3.00,60.00)	12.00(2.00,62.25)	12.00(3.00,36.50)	0.820
Days of mechanical ventilation [days, medium (IQR)]				
	0.00(0.00,0.00)	0.00(0.00,0.00)	0.00(0.00,5.00)	< 0.01
Days of physical restraints [days, medium (IQR)]				
	0.00(0.00,5.00)	0.00(0.00,4.00)	4.00(0.00,8.00)	< 0.01

### Univariate analysis

Table 1 shows the results of our univariate analysis of the categorical data. We found that gender, cognitive or motor dysfunction, developmental delay, urgent admission, metabolic acidosis, liver failure/liver dysfunction, blood transfusion, sleep deprivation, corticosteroids use, benzodiazepines use, dexmedetomidine use, barbiturates use, propofol use, anticholinergic drugs use, opioids use, vasoactive drugs use, watching television/listening to music/playing with toys(WT/LM/PT), and keeping a diary/drawing pictures(KD/DP) were each statistically significantly related to delirium. Table 2 shows the results of our univariate analysis of the continuous data. Here we found that days of mechanical ventilation and physical restraints were both statistically significantly related to delirium. However, hyperbilirubinemia, familiar items, and age were not statistically related to delirium. In the univariate analysis of admission diagnosis, there was also a statistically significant difference between each pair of subgroups ( χ^2^ = 91.975, *P* < *0.05*).

### Multivariate analysis

**Fig. 2:**
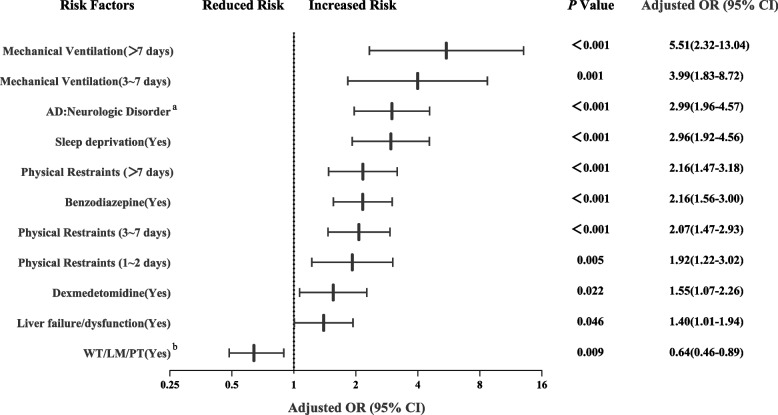
Multivariate associations with delirium. **a** Admission diagnosis: neurologic disorder, reference = all other diagnosis for admission. **b** WT/LM/PT is watching television/listening to music/playing with toys

Based on the above univariate analysis, hyperbilirubinemia, familiar items, and age were excluded for the subsequent multivariate analysis. Using logistic regression, adjusted odds ratios were used to evaluate if there were an independent relevance between development of delirium and days of mechanical ventilation, days of physical restraints, admission diagnoses of neurologic disorder, sleep deprivation, benzodiazepines use, dexmedetomidine use, liver failure/liver dysfunction, and watching television/listening to music/playing with toys (WT/LM/PT). Watching television/listening to music/playing with toys (WT/LM/PT) was the only protective factor for delirium found (Fig. 2). Among the other factors, days of mechanical ventilation had the strongest effect, with an OR of 5.51 or 3.99 if a patient had been mechanically ventilated more than 7 days or 3 to 7 days if nonmechanically ventilated, respectively.

### Patient outcomes

**Fig. 3:**
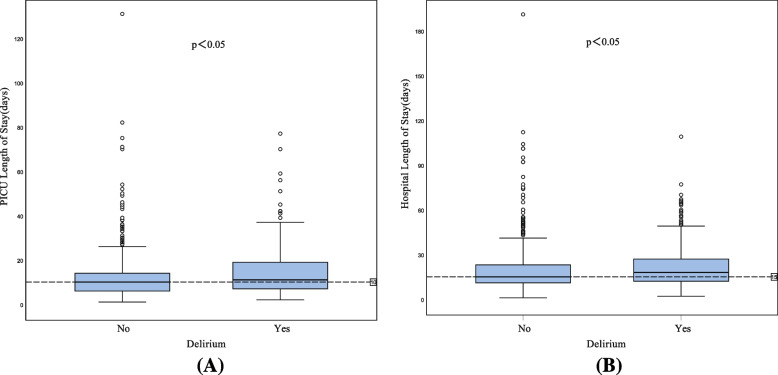
Outcomes of patients with and without delirium. **A** Length of PICU stay of patients with and without delirium. **B** Length of hospital stay of patients with and without delirium

We collected total 19,563 PICU days, and the medium length of a PICU stay was 10 days (IQR 6 to 16 days). In total there were 30,455 hospital days, with a median length of 16 days (IQR 11 to 24 days). Twenty-eight (1.8%) children died during their PICU stays. PICU lengths of stay were 11 (IQR,7.00–19.00) and 10 (IQR,6.00–14.00) days in patients with and without delirium, respectively, a difference that was statistically significant (Fig. 3A). Similarly, hospital lengths of stay were 18 (IQR,12.00–27.00) and 15 (IQR,11.00–23.00) days in patients with and without delirium, respectively, a difference that was also statistically significant (Fig. 3B). In addition, the in-hospital mortality rates were 4.63% and 0.77% in patients with and without delirium, respectively, a difference that was statistically significant as well (*P* < 0.05).

## Discussion

There have been many investigations into delirium in children to date, but there are few reports from China that have large sample sizes. To the best of our knowledge, this is the largest study on delirium in critically ill children ever conducted in China. In this single-center, prospective study involving daily assessments over 19,500 PICU days, 1,576 critically ill children underwent routine clinical delirium screening, and approximately one-quarter of them developed delirium during their PICU stay. This frequency is consistent with previous reports on delirium in critically ill children [24, 25]. We identified seven risk factors and one protective factor associated with delirium in critically ill children. Furthermore, we found that children with delirium had longer lengths of stay in both the PICU and hospital as well as a higher risk of mortality.

We found that children primarily diagnosed with a neurological disorder had the highest incidence of delirium; nearly half of these children developing delirium. Neurological disorder was also identified as one of the precipitating risk factors for delirium, and children primarily diagnosed with a neurological disorder were 2.99 times more likely to develop delirium. One of the main symptoms of a neurological disorder is consciousness dysfunction, which, coupled with decreased cognitive reserve, makes this subgroup particularly susceptible to delirium [3, 25]. Notably, distinguishing between delirium and other types of consciousness dysfunction, especially in young children, can be challenging, given that delirium is characterized by a fluctuating course of disturbed awareness and cognition. Furthermore, we found that delirium was independently associated with liver failure or dysfunction in critically ill children during their PICU stay. Hepatic encephalopathy (HE) is a characteristic of liver failure, defined as “a condition which reflects a spectrum of neuropsychiatric abnormalities seen in patients with liver dysfunction after exclusion of other known brain disease”, and delirium is often the first manifestation of HE. Moreover, the two conditions may share similar physio-pathological characteristics [26, 27], although delirium was observed 54.88 times more frequently in a previous study of critically ill children with liver failure or dysfunction [27]. However, after carefully controlling for other predictors, delirium odds were only 1.40 times higher in our study.

Sleep deprivation is a significant but often underestimated problem among critically ill children, even though good sleep is known for its healing power [28]. Yet the complex ICU environment with its invasive medical procedures, nurse care interruptions, pain, drug administration, noise, anxiety, and lack of natural circadian rhythms contributes to sleep deprivation [29]. Our results indicate that approximately 8.6% of children suffered from sleep deprivation, consistent with a related study that 6% of all hospitalized children needed prescribed medications to promote sleep [30]. Another study found that PICU patients only slept for a mean total of 4.7 h during a 10-h night, with a mean of 9.8 awakenings and a mean sleep episode length of only 27.6 min [31]. Furthermore, implementation of environmental modifications to maintain healthy sleep conditions at night (minimizing noise, light, and stimulation) may impact the occurrence rate and severity of delirium in children [32–34], and promoting circadian health may also help prevent delirium [35]. Based on the diurnal dysregulation hypothesis, sleep deprivation has long been linked to the development of delirium and other psychoses [36]. A recent study found that circadian disruption sensitizes mice to delirium due to down-regulation of hippocampal E4 promoter-binding protein [37]. Therefore, many studies recommend optimizing sleep hygiene as one of the primary strategies for managing delirium in critically ill children [18, 38–40].

Benzodiazepine exposure has already been found to be a common risk factor for pediatric delirium [41, 42], and we have drawn a similar conclusion. Reducing or avoiding deliriogenic medications including benzodiazepines is advantageous and recommended in many guidelines [18, 22, 43]. However, at present benzodiazepine is still one of the most commonly used sedative medications in Chinese PICUs [8, 44]. Seeking combined medication therapy or alternative drugs to reduce the dosage of benzodiazepine are thus needed. In addition, dexmedetomidine exposure was independently associated with delirium. However, some studies have come to completely opposite conclusions and shown that dexmedetomidine may actually help decrease the risk of delirium [45, 46]. It therefore remains unclear whether dexmedetomidine simply reduces the dose of delirium-causing drugs or confers direct neuroprotective effects. Interestingly, though, more recent findings have shown that the use of dexmedetomidine does not significantly reduce the incidence of delirium, and more adverse events have been reported in dexmedetomidine groups [47–49]. Unfortunately, we cannot suggest causality between sedatives and delirium based on our observational study design.

In addition to the factors mentioned above, we found that children watching television, listening to music, or playing with toys during their stay in the PICU were the only protective factors in this study. This could be attributed to the fact that these activities help distract children's attention from the discomfort, pain, and distress induced by diseases and unfamiliar environments, relaxing them while mitigating their anxiety. This finding has been also been reported in other studies. Playing music was one of the most commonly used nonpharmacological interventions for delirium based on a survey of pediatric cardiac intensive care units [50]. Furthermore, a pilot trial created a delirium prevention toolkit for the PICU that included developmentally appropriate toys, books, music, a DVD player, and movies, and it received favorable reviews [51].Currently, although the individual factors that are effective in preventing delirium remain unclear, nonpharmacological interventions such as watching television, listening to music, and playing with toys are still potentially helpful for the clinical management of delirium in the pediatric intensive care unit.. More high-quality randomized controlled trials are still needed, however.

Delirium was found to be associated with poor outcomes in this study. The median length of stay in both the PICU and hospital were one day and three days longer for patients with delirium compared to those without, respectively. This is consistent with previous studies conducted on critically ill children [52, 53]. In addition, in-hospital mortality was significantly higher for critically ill children with delirium in this study (5.24% vs. 0.94%, *p* < 0.001), which in accordance with the findings of another study that reported a 4.39 times higher mortality rate among delirious children [54]. Recently, more outcomes about delirium, such as total dosage of medicine, economic burden, prolonged mechanical ventilation, long-term cognitive impairment, re-admission rate, and post-intensive care syndrome have also been reported and are worth tracking in subsequent studies [55, 56].

## Limitations

Indeed, there were several limitations to this study. First, it is an observational study, so only associations can be determined and no causality. Additionally, this study was conducted in only one hospital in China, so the reported incidence of delirium may not be applicable to the country as a whole. More multi-institutional studies on delirium in PICUs are necessary. Second, we did not analyze the timeline (in PICU days) to the onset of delirium, which means that the distribution of delirium episodes during PICU stays needs further exploration. Moreover, due to insufficient human resources at night, our study was only performed during the daytime, which could have resulted in underestimating the true prevalence of delirium. Furthermore, we did not identify delirium subtypes. Finally, this study did not assess the relationship between total drug dosage and development of delirium, and other candidates including severity of illness scores, pain score, and family involvement, may have an effect on delirium as well. Further research is therefore necessary to explore more potential risk factors.

## Conclusion

Delirium is common in critically ill children in China, with a prevalence of 26%. Several risk factors for delirium development are controllable, and watching television/listening to music/playing with toys may help decrease delirium occurrence. Children with delirium had worse outcomes, and interventional studies are urgently needed.

## Data Availability

The data that support the findings of this study are available from the corresponding author upon reasonable request.
